# In Vitro Toxicity Assessment of Stilbene Extract for Its Potential Use as Antioxidant in the Wine Industry

**DOI:** 10.3390/antiox8100467

**Published:** 2019-10-09

**Authors:** Concepción Medrano-Padial, María Puerto, F. Javier Moreno, Tristan Richard, Emma Cantos-Villar, Silvia Pichardo

**Affiliations:** 1 Area of Toxicology, Faculty of Pharmacy, C/Profesor García González n°2, Universidad de Sevilla, 41012 Seville, Spain; cmpadial@us.es (C.M.-P.); mariapuerto@us.es (M.P.); 2 Area of Cellular Biology, Faculty of Biology, Avda. Reina Mercedes s/n, Universidad de Sevilla, 41012 Seville, Spain; onorato@us.es; 3 Faculté des Sciences Pharmaceutiques, Unité de Recherche OEnologie EA 4577, USC 1366 INRA, Equipe Molécules d’Intérêt Biologique (Gesvab) - Institut des Sciences de la Vigne et du Vin, Université de Bordeaux, CS 50008 - 210, Chemin de Leysotte, 33882 Villenave d’Ornon, France; tristan.richard@u-bordeaux.fr; 4 Instituto de Investigación y Formación Agraria y Pesquera (IFAPA), Centro Rancho de la Merced, Consejería de Agricultura, Ganadería, Pesca y Desarrollo Sostenible (Junta de Andalucía). Cañada de la Loba, 11471 Jerez de la Frontera, Spain; emma.cantos@juntadeandalucia.es

**Keywords:** cytotoxicity, stilbene, wine, antioxidant, Caco-2, Hep-G2

## Abstract

The reduction of sulfur dioxide in wine is a consumer’s demand, considering the allergic effects that may occur in people who are sensitive to it. Stilbenes are candidates of great interest for this purpose because of their antioxidant/antimicrobial activities and health properties, and also because they are naturally found in the grapevine. In the present study, the in vitro toxicity of an extract from grapevine shoots (with a stilbene richness of 45.4%) was assessed in two human cell lines. Significant damage was observed from 30 μg/mL after 24 h, and 40 µg/mL after 48 h of exposure. Similarly, the ultrastructural study revealed a significant impairment of cell growing. The extract was able to protect cells against an induced oxidative stress at all concentrations studied. In view of the promising results, a more exhaustive toxicological assessment of the extract is needed to confirm the safety of its further use as additive in wine.

## 1. Introduction

The most widely used preservative in the wine industry is sulfur dioxide (SO_2_) so far. Nevertheless, the exposure to SO_2_ may have health side effects such as dermatitis, urticarial, angioedema, diarrhea, abdominal pain, bronchoconstriction, and anaphylaxis [1]. Therefore, alternatives to the use of SO_2_ in wines are required. Moreover, the International Organization of Vine and Wine (OIV), in agreement with previous European Commission regulations (Ruling nº 606/2009) [2], recommended that the total SO_2_ content should not exceed 150 mg/L in red wines and 200 mg/L in white and rosé organic/conventional wines [3]. In this regard, the wine industry is developing strategies to reduce and/or replace SO_2_. The most promising tools for the replacement of SO_2_ in wine are the use of physical methods (ultrasounds, ultraviolet, pulsed electric fields, high hydrostatic pressure, etc.) and the addition of different compounds (dimethyl dicarbonate, bacteriocin, phenolic compounds, enzymes, colloidal silver complex, etc.) [1]. An alternative must be accomplished with SO_2_ action on antioxidant and antimicrobial capacity, wine oenological parameters, and organoleptic characters. However, despite the antimicrobial and antioxidant properties presented by these methods, these technologies require complex and expensive equipment. Most importantly, none of them have proven to be as effective as SO_2_ by itself so far [1,3]. For the scientific community and the wine industry, it is a challenge developing new alternatives to completely or partly eliminate the use of SO_2_ in the winemaking process to produce healthier wines but maintaining the quality requirements of consumers [4]. Consumers’ demand for natural food additives can lead more food manufacturers to substitute synthetic antioxidants for natural antioxidant compounds [5]. In this sense, the use of phenolic compounds should be highlighted due to the favorable results recently obtained. Natural extracts rich in stilbenes have been assayed for this purpose. Grape stems extracts are especially rich in flavonoids and stilbenes, with high concentrations of *trans*-resveratrol and ε-viniferin, showing high antioxidant activity and good antimicrobial properties [6,7,8,9]. Thus, wines treated with extracts obtained from grapevine shoots have reported excellent enological parameters, higher color intensity, and purity than wines treated with SO_2_, and satisfactory organoleptic features analysis [10,11]. Therefore, grapevine shoots have a promising development as a source of naturally available additives.

Despite natural additives are perceived as posing no health risk to consumers, the safety of these compounds needs to be assured before its commercialization [5]. In this concern, the toxicological studies required by the European Food Safety Authority (EFSA) comprise toxicokinetics, genotoxicity, and in vivo toxicity studies [12]. The first approach to the toxicity effect of any compound should be the in vitro cytotoxicity tests to define basal cytotoxicity, directly related to cell death induction. These studies are very useful to set the concentration range to perform further in vitro testing (genotoxicity studies) and confirm its safety to be used in the food industry [13].

In this regard, the cytotoxicity of different stilbenes have been already assayed in different cell lines. The viability of cultured macrophages, tumor-derived human T cells, and human epidermoid carcinoma cells have been reported to be inhibited by resveratrol and piceatannol in a concentration-dependent manner [14]. Vineatrol^®^ (an extract of grapevine-shoot containing resveratrol and other stilbenes) has exhibited a higher antiproliferative effect than resveratrol *per se* in various cancer cells assayed in vitro [15,16,17]. However, resveratrol and Vineatrol^®^30 exerted a concentration-dependent cytotoxic effect on V79 cells, the effect being much more pronounced with resveratrol than with Vineatrol^®^ [8]. Therefore, although the effect of some of the major stilbenes contained in the grapevine shoot extract is known, the concomitant presence of different stilbenes and other substances may modulate the individual response. As instance, Vineatrol^®^ has a different antiproliferative activity than their main compounds, with a possible synergistic effect of both resveratrol and ε-viniferin [17]. For this reason, the cytotoxicity study of the extract is mandatory considering that it is not possible to infer the summative effect of stilbenes.

In addition, it is known that stilbenes have antibacterial, antifungal, cardioprotective, neuroprotective, and pharmacological properties including antiaging effects [18,19]. While its antioxidant ability can be highlighted, stilbenes are capable of scavenging or activating cellular-enzymatic antioxidant defenses decreasing the production of intracellular reactive oxygen species (ROS) [20]. However, phenolic compounds usually exhibit both antioxidant and prooxidant activities at different doses [21,22]. Consequently, the scavenging activity of the grapevine shoot extract is studied in the present work together with the oxidative stress status and the protective and reversion properties of this extract against an oxidant agent in Caco-2 and HepG2 cell lines.

Considering the promising use of this stilbene-rich extract in the wine industry, the present work studies the cytotoxicity of the extract, including Caco-2 (colorectal adenocarcinoma cells) and HepG2 (epithelial liver cancer cells). Furthermore, an exhaustive morphological assay was carried out in order to evidence ultrastructural cellular injures that would clarify the mechanism of action of the extract. In addition, the alteration in the oxidative status and glutathione (GSH) content as well as the protective/reversion effect were investigated in both cell lines after short term exposure to this extract.

## 2. Materials and Methods

### 2.1. Supplies, Chemicals and Model Systems

Culture medium, fetal bovine serum, and cell culture reagents were provided by Gibco (Biomol, Sevilla, Spain). The rest of the chemicals were purchased from Sigma-Aldrich (Madrid, Spain) and VWR International Eurolab (Barcelona, Spain).

The human cell lines Caco-2 and HepG2 were maintained as described in Gutierrez-Praena et al. (2012) [23].

### 2.2. Stilbene-Enriched Extract

The method used for the preparation of stilbene extract was reported in a previous work [24]. Grapevine shoots were harvested in 2015 in Bordeaux region (France) and were composed of a mixture of Merlot and Cabernet Sauvignon varieties of *Vitis vinifera*. Before extraction, shoots were dried in open air for at least two months. Finely ground grapevine shoots were extracted with two times 20 L of acetone-water (6:4, *v/v*) at room temperature under agitation, twice for 12 h. After filtration, acetone was removed by evaporation under reduced pressure and the aqueous phase was lyophilized. Finally, the extract was deposited on an Amberlite XAD-7 column and washed with water. The column was then eluted with acetone. The efficiency of this process is 5.5% giving 55 g of stilbene extract per kilogram of stems.

Furthermore, the extract was fractionated by centrifugal partition chromatography (CPC) using the method of Biais et al. (2017) [7]. Briefly, the extract was eluted using the biphasic Arizona solvent system K in descending mode with the organic phase acting as stationary phase. For each run, 10 g of the extract were injected. Peak detection was monitored at 254, 280, 306, and 320 nm leading to six fractions. Only the fractions containing stilbenes were collected. The nine main stilbenes were quantified by HPLC-DAD, indicating that the stilbene-enriched extract contained at least 45.38% ± 5% of total stilbenes (*w/w*) [25]. Compounds were identified by UV spectrum and retention time from standards. *Trans*-resveratrol was quantified at 306 nm as *trans*-resveratrol; piceatannol was quantified at 320 nm as *trans*-piceid; ε-viniferin, r2-viniferin and ω-viniferin were quantified at 320 nm as ε-viniferin; hopeaphenol, isohopeaphenol, pallidol, miyabenol, and ampelopsin A were quantified at 280 nm as ampelopsin A [25].

### 2.3. Test Solutions

The range of the extract concentrations for the cytotoxicity tests was selected considering the concentration to be incorporated in wine (100 µg/mL). Serial test solutions (0–100 µg/mL) were prepared from stock solution (1000 µg/mL) in dimethyl sulfoxide (DMSO), being the final concentration in DMSO below 0.5%. 

Concentrations of the extract used for both oxidative stress assays were calculated based on the cytotoxicity study previously performed. The mean effective concentration (EC_50_) value obtained (31.18 µg/mL and 20.56 µg/mL in HepG2, and 55.77 µg/mL and 39.02 µg/mL in Caco-2 cells for 24 h and 48 h, respectively) was chosen as the highest exposure concentration, along with the fractions EC_50/2_ and EC_50/4_. 

To measure protection and reversion abilities, test solutions of the extract to cell viability greater than 75% were selected (EC_50/2_ and EC_50/4_).

### 2.4. Cytotoxicity Assays

The exposure concentrations were prepared in medium, with the highest concentration being 100 µg/mL. For the control group culture medium without the extract was used. Moreover, a control of solvent (0.5% of DMSO) has also been included. The exposure concentrations were added to both cells and the cytotoxicity assays were performed after 24 and 48 h of exposure. 

Neutral red uptake (NR) was evaluated according to Borenfreund and Puerner (1984) [26] with modifications [27]. MTS (3-(4,5-dimethylthiazol-2-yl)-5-(3-carboxymethoxyphenyl)-2-(4-sulfophenyl)-2H- tetrazolium salt) reduction was measured according to the procedure of Baltrop et al. (1991) [28]. Moreover, total protein content (TP) was performed according to the procedure described by Bradford (1976) [29]. 

### 2.5. Morphological Study under Light and Transmission Electron Microscope

Light and electron microscope observations were performed according to Gutiérrez-Praena et al. (2019) [30]. Cultured cells were exposed to three different concentrations of the extract, the EC_50_ value, and their fractions (EC_50_/2, EC_50_/4). HepG2 were exposed to 7.79, 15.59 and 31.18 µg/mL and Caco-2 cells were exposed to 13.90, 27.88, and 55.77 µg/mL.

### 2.6. Oxidative Stress and Antioxidant Ability Assays

The oxidative stress endpoints measured, ROS content and GSH levels, were carried out following the methods described in Gutiérrez-Praena et al. (2012) [23]. The results of both assays were expressed as relative light units (RLU).

For the estimation of the protection and reversion abilities of extract, H_2_O_2_ 100 µM was administered to induced changes in the cell membranes and antioxidant system in HepG2 and Caco-2 cells [31].

For the protection assay, after discarding the previous medium, exposure solutions (EC_50/2_ and EC_50/4_) of the extract were first added to the cells and incubated at 37 °C for 24 h or 48 h. After the treatment time, the medium was discarded and then exposed to 100 µM H_2_O_2_ for 2 h. Similarly, Caco-2 and HepG2 were pre-treated with H_2_O_2_ for 2 h for the reversion assay and a later exposure of the extract for 24 or 48 h. Unexposed cells were included in the figures in order to compare the results with basal levels of ROS and GSH. A control of DMSO was also incorporated in all plates.

Then, both abilities were evaluated by measuring the ROS levels and GSH content as previously described.

### 2.7. Calculations and Statistical Analysis

All experiments were performed three times per assay. The data for all experiments were given as the arithmetic mean percentage ± standard deviation in relation to control. Statistical analysis was performed using analysis of variance (ANOVA) followed by Dunnett’s multiple comparison tests. Differences were considered significant in respect to the control group at *p* < 0.05 (*), *p* < 0.01 (**) and at *p* < 0.001 (***).

## 3. Results

### 3.1. Presence of Stilbenes in the Extract

The main compounds were ε-viniferin (16.34%, *w/w*), *trans*-resveratrol (8.07%, *w/w*), isohopeaphenol (4.11%, *w/w*), ampelopsin A (3.21%, *w/w*), pallidol (3.12%, *w/w*), ω-viniferin (2.77 %, *w/w*), miyabenol C (2.75%, *w/w*), r2-viniferin (2.24%, *w/w*), hopeaphenol (2.01%, *w/w*), and piceatannol (0.76%, *w/w*). The stilbene-enriched extract contained 45.38% (*w/w*) of total stilbenes.

### 3.2. Cytotoxicity Studies

**Figure 1:**
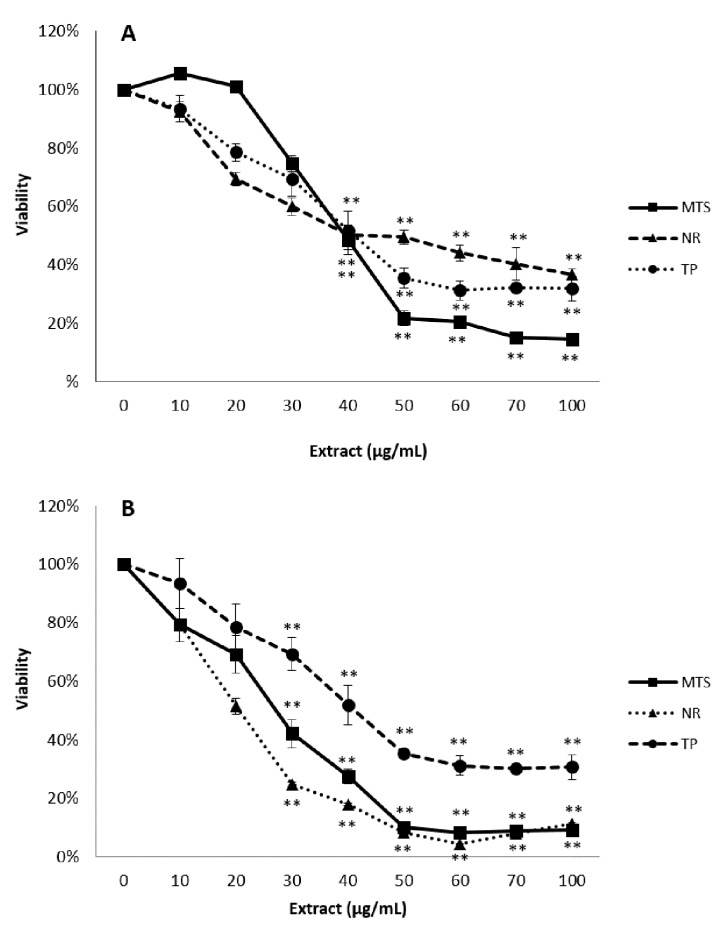
Reduction of tetrazolium salt (MTS), neutral rep uptake (NR), and total protein content (TP) of HepG2 cells exposed for 24 h (**A**) and 48 h (**B**) to 0–100 µg/mL of the stilbene extract (45%). All values expressed as mean ± SD. Significant differences in respect to the control from *p* < 0.01 (**).

Both cell types exposed to the extract underwent a concentration and time-dependent decrease in all endpoints. In HepG2 cells, after 24 h of exposure, the MTS assay indicated a significant reduction in the cellular viability at concentrations of 40 µg/mL and above, showing greater alteration than the TP and NR assays (Figure 1A). Similarly, the most sensitive endpoints after 48 h were MTS reduction and NR uptake (Figure 1B). Considering the EC_50_ values, toxic effects were more evident in HepG2 cells in the longer exposure time, being 31.2 ± 2.4 µg/mL for 24 h, and 20.6 ±2.7 µg/mL for 48 h of exposure.

**Figure 2:**
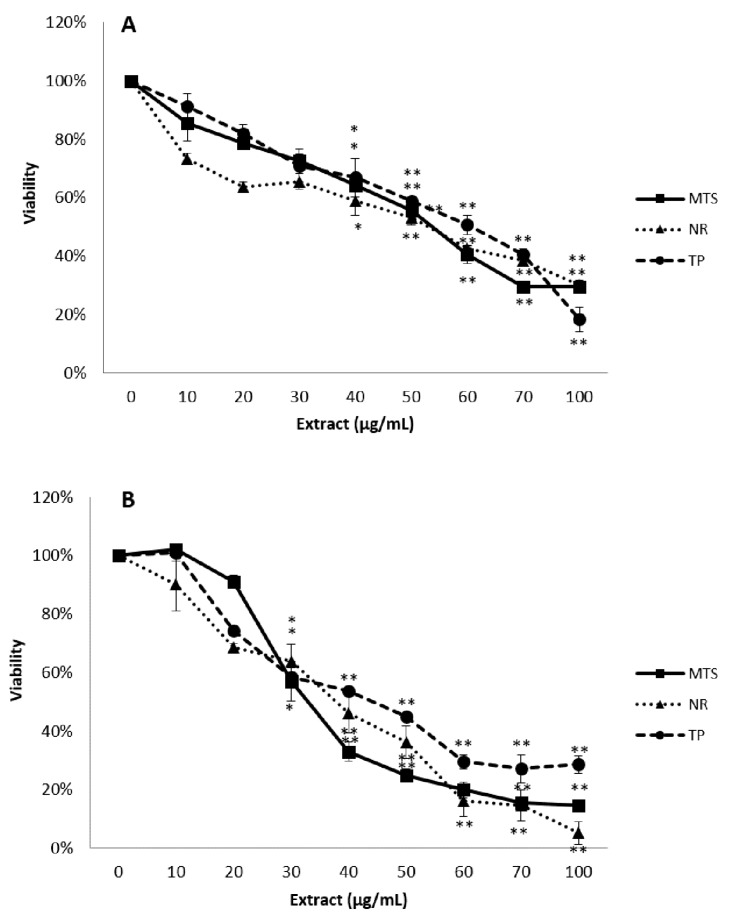
Reduction of tetrazolium salt (MTS), neutral rep uptake (NR), and total protein content (TP) of Caco-2 cells exposed for 24 h (**A**) and 48 h (**B**) to 0–100 µg/mL of the stilbene extract (45%). All values expressed as mean ± SD. Significant differences in respect to the control from *p* < 0.05 (*) and *p* < 0.01 (**).

After the exposure of Caco-2 cells to the extract, all assays showed similar concentration-dependent reduction in cell viability. MTS assay indicated a marked reduction in the cellular viability, being the EC_50_ value in this endpoint of 55.8 ± 4.0 µg/mL and 39.0 ± 2.7 µg/mL after 24 h and 48 h, respectively. These decreases were significantly different from the control group at the concentration 40 µg/mL and above at 24h, but when cells were exposed to the extract for two days, the cell viability was significantly reduced from 30 µg/mL (Figure 2).

### 3.3. Light and Electron Microscopic Observation in HepG2 and Caco-2 Cells

HepG2 and Caco-2 cells exposed to different concentrations of the extract experienced a significant impairment of cell growing which compromised the survival of the cells at high concentrations. 

**Figure 3:**
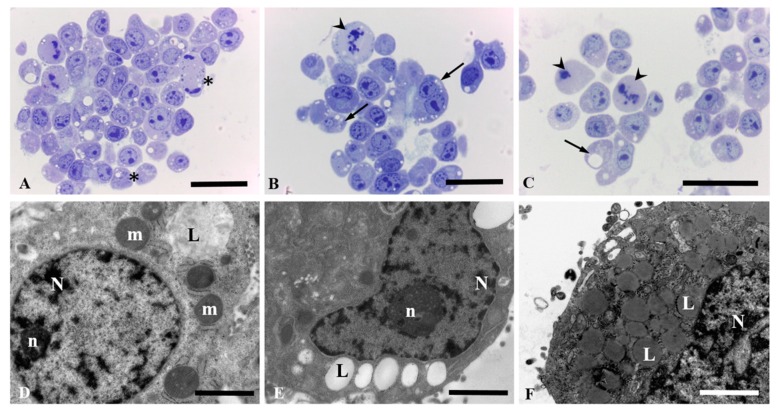
Morphology of HepG2 cells after 24 h of exposure to the extract observed by light microscopy (**A**–**C**, bars = 25 µm) and electron microscopy (**D**–**F**, bars = 2 µm). Unexposed control cultures (**A**) and HepG2 cells exposed to 7.79 µg/mL of the stilbene extract (45%) (**D**), 15.59 µg/mL of the extract (**B**,**E**) and 31.18 µg/mL of the stilbene extract (45%) (**C**,**F**). (**A**) Unexposed cells undergoing mitotic processes (arrow heads). (**B**) and (**C**) Lipid degeneration with confluent lipid drops (arrow) and aberrant mitotic figures (arrow head). (**D**) Cells showing nucleus (N) and nucleolus (n); mitochondria (m); lipid drops (L). (**E**) Big lipid drops (L); nucleus (N) and nucleolus (n) are also observed. (**F**) High amount of lipid drops that tends to confluency (L).

Under light microscope, unexposed HepG2 cells underwent normal mitotic processes (Figure 3A). However, when cells were treated with 15.6 and 31.2 µg/mL of the extract they revealed an intense lipid degeneration in the cytoplasm with vacuoles that tends to confluency (Figure 3B,C). Moreover, aberrant mitotic figures were also detected, which suggest that the extract was able to stop the cell growing at any step, including mitosis (Figure 3B,C).These morphological features were also observed under electron microscopy with cells showing big lipid drops (Figure 3D–F). 

**Figure 4:**
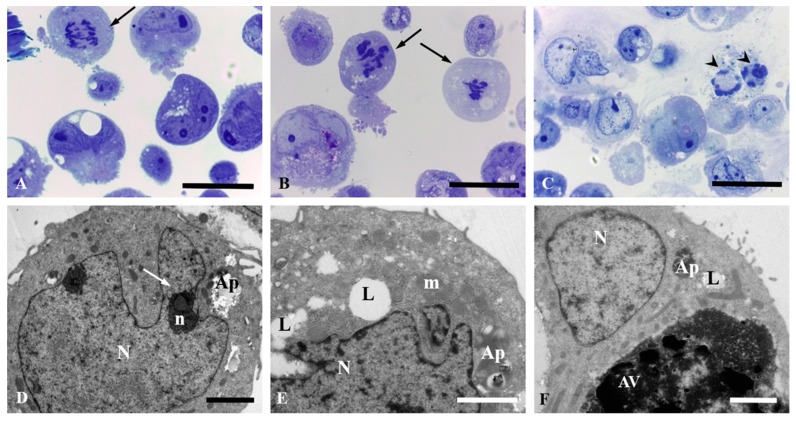
Morphology of Caco-2cells after 24 h of exposure to the stilbene extract (45%) observed by light microscopy (**A**–**C**, bars = 25 µm) and electron microscopy (**D**–**F**, bars = 2 µm). Unexposed control cultures (**A**,**D**), and Caco-2 cells exposed to 13.9 µg/mL of the extract (**B**,**E**) and 27.88 µg/mL of the extract (**C**,**F**). (**A**) Unexposed cells undergoing mitotic processes (black arrow). (**B**) Cells undergoing aberrant mitosis (black arrow). (**C**) Cells showing apoptotic nuclei condensation (arrow head). (**D**) Unexposed cells showing normal nucleus with an irregular surface (N), nucleolus (n) with large fibrillar center (white arrow), and autophagosomes (Ap) in the cytoplasm. (**E**) Damaged nucleus and mitochondria are observed as well as big lipid drops. Autophagosomes (Ap) evidenced degenerative process. (**F**) Lipid drops (L), autophagosomes (Ap), and autophagic vacuoles (AV) are shown.

The damage observed in Caco-2 cells exposed to the extract was less profuse in comparison to HepG2 cells, although a marked vacuolization was shown as well as cell death. In fact, the presence of apoptotic nuclei was more frequent in the case of Caco-2 cells. Whereas cells underwent normal mitotic process in the control group (Figure 4A), aberrant mitosis can be observed from the lowest concentration assayed (13.9 µg/mL) (Figure 4B) showing the first stages in the process of apoptosis characterized by a continuous ring of condensed chromatin at the interior surface of the nuclear envelope (Figure 4C). Caco-2 cells were analyzed by transmission electron microscopy showing a nucleus with an irregular surface, decondensed chromatin, and very developed nucleoli with well-known fibrillar center. Autophagosomal vacuoles were observed in the cytoplasm (Figure 4D). Similar findings were observed in the ultrastructural study, with the lowest concentration showing lipid degeneration (Figure 4E,F). In addition, Caco-2 cells showed big citoplasmatic inclusions as a result of autophagic processes that lead to cell death (Figure 4E,F).

### 3.4. Oxidative Stress Assays

**Figure 5:**
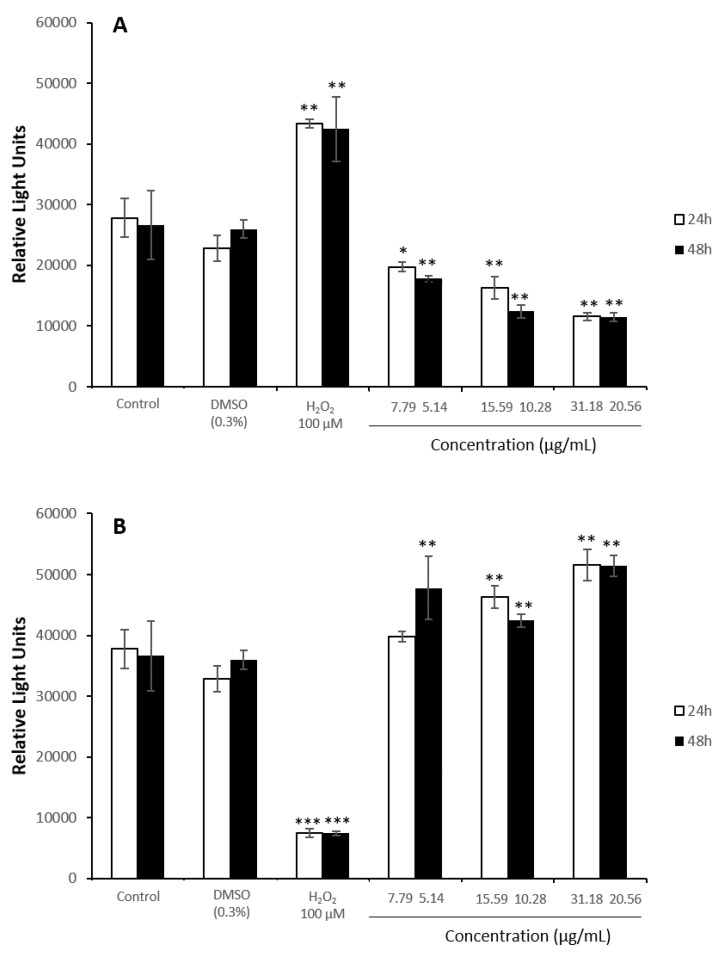
ROS content (**A**) and GSH content (**B**) in HepG2 cells exposed to 0–31.18 µg/mL or 0–20.56 µg/mL stilbene extract (45%) during 24 h or 48 h, respectively. All values are expressed as mean ± SD. Differences were considered significant compared to the control group from *p* < 0.05 (*), *p* < 0.01 (**) and *p* < 0.001 (***).

The control of solvent evidenced no significant changes when cells were exposed to 0.3% of DMSO. However, HepG2 cells experienced a significant decrease in ROS levels when they were exposed to all the concentrations of the extract tested after 24 and 48 h, showing a greater decrease after the exposure to 20.6 µg/mL (Figure 5A). Moreover, GSH content underwent concentration-dependent enhancements in comparison to the negative control group after both times of exposure (Figure 5B). 

**Figure 6:**
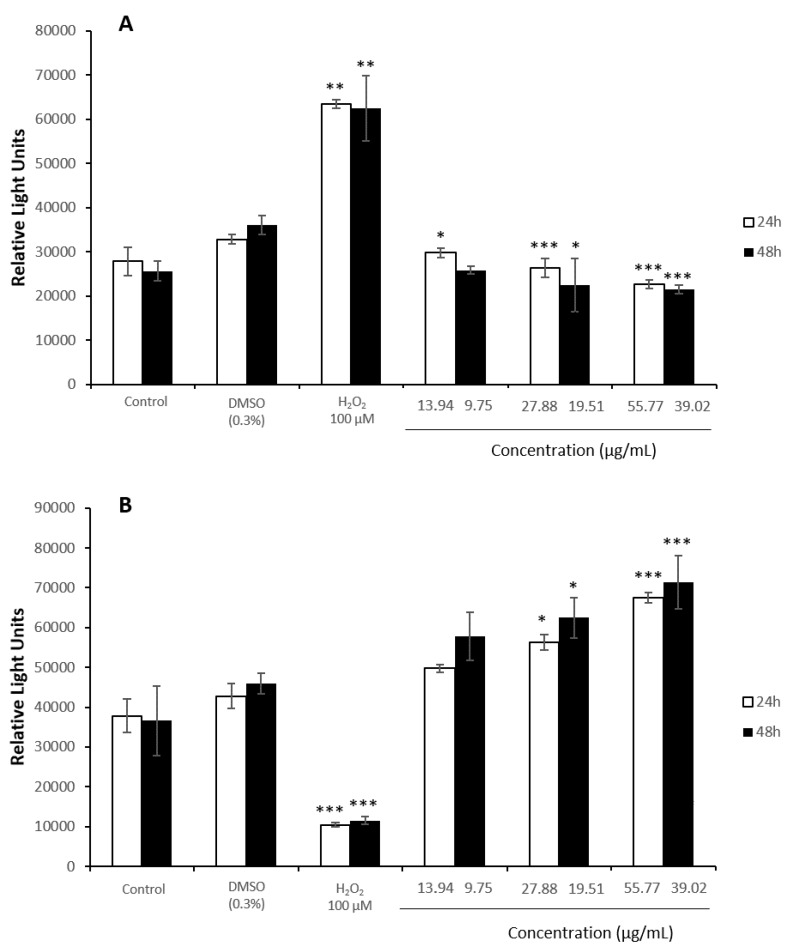
ROS content (**A**) and GSH content (**B**) in Caco-2 cells exposed to 0–55.77 µg/mL or 0–39.02 µg/mL stilbene extract (45%) during 24 h or 48 h, respectively. All values are expressed as mean ± SD. Differences were considered significant compared to the control group from *p* < 0.05 (*), *p* < 0.01 (**) and *p* < 0.001 (***).

When Caco-2 cells were exposed to increasing concentrations of the extract, ROS content was significantly reduced (Figure 6A). GSH activity was increased significantly at the highest concentrations assayed, 55.77 µg/mL and 39.02 µg/mL for 24 and 48 h, respectively, with enhancements of 1.6 folds compared to the control negative group (Figure 6B).

### 3.5. Antioxidant Assays

The antioxidant ability of the extract was evaluated taking into account their capacity to protect the cells against a further exposure of H_2_O_2_ or their capacity to revert the damage induced by this substance after a previous exposure by measuring both ROS and GSH levels. No significant changes were recorded when cells were exposed to 0.3% of DMSO (data not shown).

**Figure 7:**
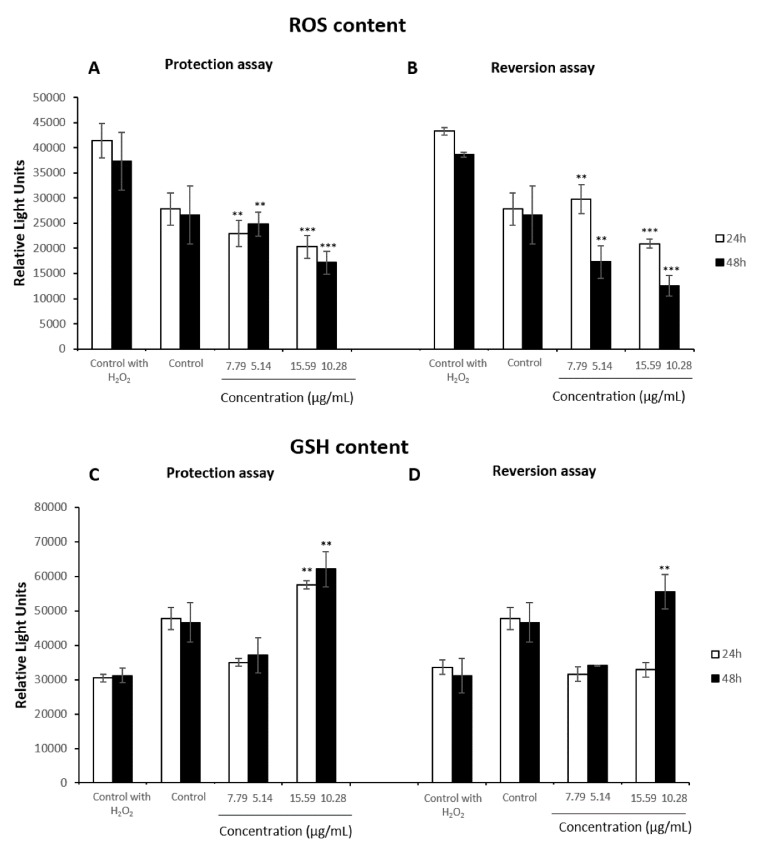
ROS (**A**) and GSH content (**C**) in HepG2 cells first pretreated with 0–15.59 µg/mL or with 0–10.28 µg/mL of the stilbene extract (45%) for 24 h or 48 h, respectively, and a later exposure to H_2_O_2_ 100 µM for 2 h. ROS (**B**) and GSH (**D**) content in HepG2 cells exposed to H_2_O_2_ 100 µM first and a later 24 h or 48 h-treatment with 0–15.59 µg/mL or 0–10.28 µg/mL, respectively. Cells exposed to H_2_O_2_ 100 µM 2 h were used as control. All values are expressed as mean ± SD. Differences were considered significant compared to the control group from *p* < 0.01 (**) and *p* < 0.001 (***).

The results showed that the extract was able to protect HepG2 cells against an induced oxidative stress at all concentrations studied, showing a marked decrease of ROS content at both treatment times (Figure 7A). Similarly, after the pre-treatment with H_2_O_2_ for 2 h, the extract presented a greater reversion role in a concentration and time-dependent manner. This reduced ROS content even lower than basal levels at the highest concentrations tested for 24 h and after 48 h of exposure of all studied concentrations (Figure 7B). By contrast, in both protection and reversion assays, GSH levels of the hepatic cells were not affected by the administration of 7.8 µg/mL during 24 h and 5.1 µg/mL for 48 h of the extract, while they experienced a significant increase when they were exposed to the highest concentration (Figure 7C,D). After the pre-treatment with 15.6 µg/mL and 10.3 µg/mL during 24 h and 48 h respectively and a later exposure of H_2_O_2_, the results showed higher GSH levels than basal content.

**Figure 8:**
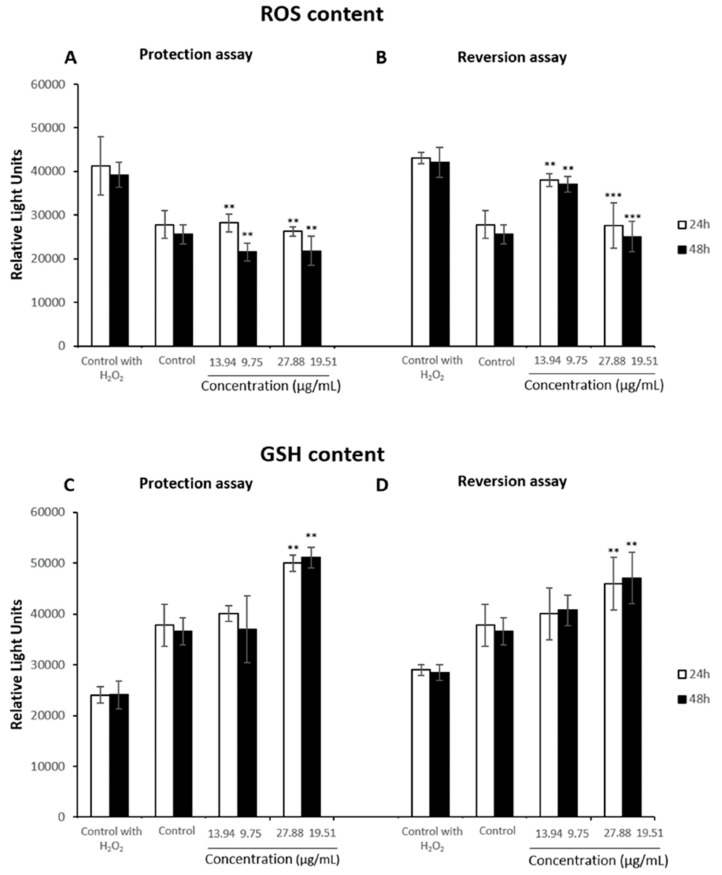
ROS (**A**) and GSH content (**C**) in Caco-2 cells first pretreated with 0–27.88 µg/mL or with 0–19.51 µg/mL of the extract for 24 h or 48 h, respectively, and a later exposure to H_2_O_2_ 100 µM for 2 h. ROS (**B**) and GSH (**D**) content in HepG2 cells exposed to H_2_O_2_ 100 µM first and a later 24 h or 48 h-treatment with 0–27.88 µg/mL or 0–19.51 µg/mL respectively. Cells exposed to H_2_O_2_ 100 µM 2h were used as control. All values are expressed as mean ± SD. Differences were considered significant compared to the control group from *p* < 0.01 (**) and *p* < 0.001 (***).

The cell line Caco-2 presented lower protection and reversion capacity when compared to the effects observed in HepG2 cells. In the protection assay, the extract was able to significantly reduced ROS content with respect to the control group treated with H_2_O_2_ at all concentrations assayed after both pre-treatment times (Figure 8A). Similar results were obtained when Caco-2 cells were exposed to H_2_O_2_ prior the extract (Figure 8B). At the highest concentrations assayed ROS content was reduced down to basal levels. With respect to GSH levels, in both reversion and protection assays, GSH content were higher than basal levels after both times of treatment at the highest concentration assayed. The results showed a significant increase between control group with H_2_O_2_ treatment and those exposed to the highest concentration tested of the extract for 24 h and 48 h prior H_2_O_2_ (Figure 8C). Moreover, after the exposure to H_2_O_2_ for 2 h, only Caco-2 cells treated with 22.9 µg/mL during 24h or with 19.5 µg/mL for 48 h presented a significant increase of GSH levels (Figure 8D).

## 4. Discussion

Previous reports have revealed the interesting properties of stilbene-rich extracts form grapevine-shoot to be used in the vine and wine industry [3,10,23,32]. Therefore, the cytotoxicity study in human cell lines (Caco-2 and HepG2) is a key step in order to evaluate its safety. The cytotoxicity study performed in the present work evidenced that our extract (45% of stilbenes) reduced cell viability from 40 µg/mL after 24 h of exposure and from 30 µg/mL at 48 h in both cell lines. Similarly, Vineatrol^®^ (29% of stilbenes) inhibits cell proliferation in another human colon carcinoma cell line (SW480 cells), showing a concentration and time-dependent reduction in the MTT assay [16]. The latter extract also affected cell cycle progression of different human cancer cell lines (SW480, SW620, and HTC 116), making them more prone to suffer cytotoxic effects of toxicants [33]. Moreover, these authors have previously reported that Vineatrol^®^ disrupts cells proliferation more efficiently than resveratrol on HepG2 cells [17].

Studies using single stilbenes are more numerous in comparison to those using stilbenes extracts. In this sense, a concentration-dependent cytotoxicity of resveratrol and piceatannol have been reported in cultured macrophages, tumor-derived human T cells, and human epidermoid carcinoma cells [14]. These results showed cell inhibition at high concentration of resveratrol (50 µmol/L) in all cell types, while at low concentration (5 µmol/L) stimulation of macrophages was detected. Moreover, Colin et al. (2009) reported that the mixture of *trans*-resveratrol and ε-viniferin (Vineatrol^®^) as well as resveratrol itself affect cell cycle progression of human colon cancer cell lines [33]. Similarly, both resveratrol and Vineatrol^®^ exerted a concentration-dependent cytotoxic effect on V79 cells, the effect being much more pronounced with resveratrol than with Vineatrol^®^ [8]. In addition, Mizuno et al., 2017 reported that different modifications of stilbenes have different cytotoxic effect in CHO-K1 and HepG2 cells [34]. The latter authors hypothesized that the cell viability decrease may be reduced because of the appearance of necrosis or late stages of apoptosis when the metabolic activity is harshly reduced. This finding has been observed in the morphological study carried out in the present work, showing the first stages of apoptosis such as condensed chromatin in the nuclear envelope in Caco-2 cells exposed to the stilbene extract. Similarly, nuclear staining of the human colorectal cells SW480, evidenced that resveratrol-analogs and Vineatrol^®^ preparation caused a nuclear redistribution of cyclin A, could be the first step for apoptosis considering that this finding was observed in both normal and apoptotic cells [33]. SW480 cells experienced apoptosis in a dose and time-dependent way. Moreover, the resveratrol tetramer, r-viniferin, provoked a cell cycle arrest followed by apoptosis in the prostate cancer cell line LNCaP, being this effect higher than in the exposure to resveratrol where apoptosis was not observed [35]. Also, different cultures of leukemic cells from chronic B cell malignancies and normal peripheral blood-derived mononuclear cells were exposed to Vineatrol^®^. Whereas impairment of cell proliferation and apoptotic processes were observed in exposed leukemic cells, the survival of normal peripheral blood mononuclear cells was little affected in the presence of these polyphenolic compounds and higher concentrations were required in order to elicit cell death [15]. This finding is particularly interesting since normal cells are usually more sensitive than cancer cell lines, highlighting the anti-cancer effect of stilbenes [36,37]. Ji et al. (2018) reported that cancer cells observed under microscope became flattened and elongated, which were morphological changes of cellular senescence [38]. In addition, resveratrol arrested cancer cell proliferation in a concentration-dependent way. However, no effect was recorded in cell viability for non-tumorigenic human breast epithelial cells MCF-10A. In this regards, Elshaer et al. (2018) reviewed the anti-cancer mechanisms of resveratrol by inducing autophagy and apoptosis [39]. In addition to death cell and autophagy, other morphological changes were observed in cells exposed to stilbenes. Scanning electron microscopy revealed that resveratrol altered the morphology of T cells and skin cells at concentrations ≥50 μmol/L, especially affecting the cell membrane [14]. Besides cell death, previous degradation steps have been evidenced in the present work. Hence, one of the most remarkable morphological features were presence of big lipid drops evidencing lipid degeneration, probably due to the degenerative processes in the organelles [40].

The defense against oxidative damage is one of the most commonly described characteristics of stilbenes [21]. However, phenolic compounds frequently exhibit prooxidant or antioxidant activities depending on the dose, alkali pH, high concentrations of transition metal ions, and the presence of oxygen molecules [21,41,42]. In this regard, Cotoras et al., (2014) assessed in vitro and in vivo antioxidant and prooxidant properties of phenolic compounds obtained from *Vitis vinifera* pomace, evidencing a prooxidant effect by accumulation of ROS [43]. By contrast, our results suggest that the extract modulates important functions related to the maintenance of Caco-2 and HepG2 redox environment, preventing the increase in ROS levels induced by H_2_O_2_, and even reverting them down to basal levels, but no prooxidant effect was recorded. Goutzourelas et al. (2015) also found that two extracts from stems of Greek grape varieties significantly decreased the ROS levels by 21.8 ± 2.0% and 16.5 ± 3.7%, respectively, compared to the control group, in C2C12 cells after 24 h of exposure [44]. This antioxidant activity can be explained by the ability of polyphenols to enhance antioxidant mechanisms, such as GSH synthesis, which acts as a scavenger to directly remove hydroxyl radical and singlet oxygen and participates in the elimination of hydrogen peroxide. In addition, Müller et al. (2009) found that the grapevine-shoot extract Vineatrol^®^30, acted as a free radical scavenger and potent antioxidant enhancing the glutathione peroxidase activity at non-cytotoxic concentrations in V79 Chinese hamster lung fibroblast cells [8]. Similarly, an increase of GSH levels in endothelial cells was observed after 24 h of exposure of polyphenolic extracts derived from the stems of three Greek grape varieties [45].

## 5. Conclusions

Besides all beneficial abilities of stilbenes, such as antioxidant activity, they may also have potential toxic effects. In this regard, the present work entail the first approach to the toxicological studies required before being used in wines. The cytotoxicity study evidenced that the stilbene extract reduced cell viability in both cell lines in the range of concentrations from 40–100 µg/mL after 24 h of exposure and from 30–100 µg/mL at 48 h of exposure. In addition, ultrastructural changes were observed from 15.6 µg/mL in HepG2 cells and 27.9 µg/mL in Caco-2 cells, which highlighted the higher sensitivity of the morphological study in comparison to the cytotoxicity endpoints. Moreover, this work also presents a wide assessment of the prooxidant and antioxidant profile of our extract in human cells, which had not been previously studied. In general, at the exposed concentrations, the extract presented a protective and reductive role against an induced oxidative stress. Taking into account all these findings as a starting approach, more research is needed to establish effective and safe concentrations of this stilbene extract intended to be used in wines as a preservative.
